# Plant-Derived Essential Oils; Their Larvicidal Properties and Potential Application for Control of Mosquito-Borne Diseases

**DOI:** 10.31661/gmj.v8i0.1532

**Published:** 2019-08-16

**Authors:** Mahmoud Osanloo, Mohammad Mehdi Sedaghat, Alireza Sanei-Dehkordi, Amir Amani

**Affiliations:** ^1^Department of Medical Nanotechnology, School of Advanced Technologies in Medicine, Fasa University of Medical Sciences, Fasa, Iran; ^2^Noncommunicable Diseases Research Center, Fasa University of Medical Sciences, Fasa, Iran; ^3^Department of Medical Entomology and Vector Control, School of Public Health, Tehran University of Medical Sciences, Tehran, Iran; ^4^Department of Medical Entomology and Vector Control, School of Health, Hormozgan University of Medical Sciences, Bandar Abbas, Iran; ^5^Natural Products and Medicinal Plants Research Center, North Khorasan University of Medical Sciences, Bojnurd, Iran; ^6^Medical Biomaterials Research Center (MBRC), Tehran University of Medical Sciences, Tehran, Iran

**Keywords:** Volatile Oil, Pesticides, *Aedes*, *Anopheles*, *Culex*

## Abstract

Mosquito-borne diseases are currently considered as important threats to human health in subtropical and tropical regions. Resistance to synthetic larvicides in different species of mosquitoes, as well as environmental pollution, are the most common adverse effects of excessive use of such agents. Plant-derived essential oils (EOs) with various chemical entities have a lower chance of developing resistance. So far, no proper classification based on lethal concentration at 50% (LC_50_) has been made for the larvicidal activity of EOs against different species of *Aedes*, *Anopheles* and *Culex* mosquitoes. To better understand the problem, a summary of the most common mosquito-borne diseases have been made. Related articles were gathered, and required information such as scientific name, used part(s) of plant, target species and LC_50_ values were extracted. 411 LC_50_ values were found about the larvicidal activity of EOs against different species of mosquitoes. Depending on the obtained results in each species, LC_50_ values were summarized as follows: 24 EOs with LC_50_ < 10 µg/mL, 149 EOs with LC_50_ in range of 10- 50 µg/mL, 143 EOs having LC_50_ within 50- 100 µg/mL and 95 EOs showing LC_50_ > 100 µg/mL. EOs of *Callitris glaucophylla* and *Piper betle* against *Ae. aegypti*, *Tagetes minuta* against *An. gambiae*, and *Cananga odorata* against *Cx. quinquefasciatus* and *An. dirus* having LC_50_ of ~ 1 µg/mL were potentially comparable to synthetic larvicides. It appears that these plants could be considered as candidates for botanical larvicides.

## Introduction

Arthropod-borne diseases are the cause of more than 17% of all human infectious diseases around the world [1]. Mosquitoes (Diptera: Culicidae) are an important family of Arthropoda phylum which is grouped into 39 genera with a total of over 3000 species [2, 3]. More than half the world’s population lives in areas where mosquito-borne diseases are common. Mosquito-borne diseases represent a critical threat for billions of people worldwide, e.g., more than 3.9 billion people in over 128 countries are at risk of dengue, with 96 million cases estimated per year. Malaria causes more than 400,000 deaths every year globally; the majority of them are children under five years of age [1, 4]. Three genera of mosquito which are very important in the transmission of human diseases include *Aedes* (Chikungunya, Dengue fever, Lymphatic filariasis, Rift Valley fever, Yellow fever, Zika), *Anopheles* (Malaria, Lymphatic filariasis) and *Culex* (Japanese encephalitis, Lymphatic filariasis, West Nile fever) [1, 5]. All mosquitoes have immature aquatic stages. Thus, larviciding could be an efficient method to reduce the population of mosquitos and prevent the transmission of such diseases [6-8]. Larvicides reduce their population in breeding places, where they are concentrated, immobilized and accessible before they emerge into adults [9, 10]. Larviciding is usually performed by applying synthetic larvicides such as organophosphates (e.g., temephos, fenthion, and malathion) or using an insect growth regulator (IGRs) such as methoprene [11, 12]. However, indiscriminate use of these agents affects the population of their natural enemies (such as *Gambosia* fish) and causes resistance in different species of mosquitoes [10, 13]. Additionally, synthetic insecticides are usually based on a single active ingredient. Thus, resistance against them is more probable compared with botanical insecticides having multiple components [14-16]. Developing resistance against insecticides also has been linked to their tendency to remain in the environment for a long time. During this period, larva starts to produce detoxifying enzymes or change their enzymes’ structure. Thus, resistance against the larvicides may be expected [17, 18]. Moreover, synthetic insecticides leave toxic residues in the environment and make safety concerns [13, 19]. In this regards, identification of active and eco-friendly bio-pesticides is crucial for successful management of mosquito-borne diseases. Essential oils (EOs) have been suggested as alternative sources for control of insects as selective and biodegradable agents with minimal impacts on non-target organisms and environment [13, 20]. EOs are complex mixtures of volatile organic compounds which are produced as secondary metabolites in plants [21]. They are obtained from hydrodistillation or steam distillation of plant entities such as flowers, roots, barks, leaves, seeds, peels, fruits, and woods [22]. EO-based pesticides consist of a combination of molecules which can act concertedly on both behavioral and physiological processes. Thus, there is very little chance of resistance development among the treated mosquitoes [10, 21, 23]. Generally, EOs have different larvicidal activity (LA) against various species of mosquitoes. The most critical factor in developing EO-based larvicides is their potency in terms of their LAs. Currently, there is a single review paper, which has gathered LA of 122 plants against mosquitoes. However, the authors have not separated the LA-based on the mosquito species [24]. In this review we have given an update to the potential of herbal larvicides, gathering data for more than 400 LC_50_ values of EOs. EOs have been arranged based on their LC_50_, against each species to provide a better understanding and comprehensive knowledge about their larvicide potential.

## Common Mosquito-Borne Diseases

In Table-1, profiles of the most common mosquito-borne diseases (including vectors, pathogenic agent, common hosts in vertebrate and distribution) have been summarized. Malaria, Yellow Fever, Dengue Fever, Zika, Chikungunya, West Nile, and Japanese encephalitis accounted for almost 0.7 million deaths around the world, annually [1].

**Table 1:** Profiles of the Most Common Mosquito-Borne Diseases

**Disease**	**Vectors**	**Caused by**	**Vertebrate Hosts**	**Distribution**
Malaria [25]	*An. atroparvus,* *An. labranchiae,* *An. messeae, An. sacharovi, An. sergentii,* * An. superpictus Grassi,* *An. arabiensis ,* *An. funestus, An. gambiae, An. melas, An. merus,* * An. moucheti, An. nili,* * An. barbirostris,* * An. lesteri, An. sinensis,* * An. aconitus, An. annularis, An. balabacensis,* *An. culicifacies, An. dirus, An. farauti, An. flavirostris, An. fluviatilis, An. koliensis, An. leucosphyrus,* *An. maculatus, An. minimus, An. punctulatus,* * An. stephensi,* *An. subpictus, An. sundaicus.*	Protozoan parasite; Plasmodium	Reptiles, birds, rodents, Primates and humans.	Endemic throughout most of the tropics. Ninety-five countries and territories have ongoing transmission
Yellow Fever [26]	*Ae. aegypti, Ae. africanus, Ae. aromeliae,* *Ae. albopictus,* *Ae. furcifertaylori,* *Ae. luteocephalus,* * Ae. metallicus,* *Ae. bromeliae, Ae. serratus.*	Virus of the family Flaviviridae; genus Flavivirus.	Primates	Ghana, Guinea, Nigeria, Ethiopia, Liberia, Gambia, Mali, Senegal, Sudan, Togo, Uganda, Congo, Chad, Angola, Brazil, Colombia and Peru, Paraguay, Argentina,
Dengue Fever [27]	*Ae. aegypti, Ae. albopictus, Ae. polynesiensis,* *Ae. scutellaris.*	Virus of the family Flaviviridae; genus Flavivirus.	Primates	Philippines, Thailand, China, Malaysia, Japan, Pakistan, Taiwan, India, Sri Lanka, Burma, Malay Peninsula, Cambodia, Vietnam, Indonesia, India, Australia, Brazil, Venezuela, Mexico, Bolivia, Argentina, USA
Zika [28, 29]	*Ae. africanus,* *Ae. luteocephalus,* *Ae. aegypti, Ae. albopictus, Ae. furcifer, Ae. vittatus.*	The virus of the family Flaviviridae; genus Flavivirus.	Primates	Brazil, Colombia, Venezuela, Puerto Rico, Martinique, Honduras, Guadeloupe, El Salvador, French Guiana, Guinea Bissau, Angola, Cabo Verde, Thailand, Vietnam, Singapore
Chikungunya [30, 31]	*Ae. albopictus, Ae. aegypti, Ae. henselli*	Virus of the family Togaviridae; genus Alphavirus	Primates, birds, cattle, and rodents	Benin, Burundi, Cameroon, Central African Republic, Comoros, Congo, Equatorial Guinea, Guinea, Kenya, Madagascar, Malawi, Mauritius, Mayotte, Nigeria, Senegal, South Africa, Sudan, Tanzania, Uganda, Zimbabwe, Cambodia, East Timor, India, Indonesia, Laos, Malaysia, Maldives, Myanmar, Pakistan, Philippines, Réunion, Seychelles, Singapore, Taiwan, Thailand and Vietnam.
West Nile [32]	*Ae. aegypti, Cx. pipiens,* *Cx. quinquefasciatus,* *Cx. australicus,* * Cx. globcoxitus,* *Cx. tarsalis, Cx. univittatus, Cx. annulirostris*	Virus of the family Flaviviridae; genus Flavivirus.	Birds, Horses, Other Mammals	Commonly found in Africa, Europe, the Middle East, North America, and West Asia.
Japanese Encephalitis [33]	*Cx. tritaeniorhynchus,* *Cx. annulirostris*, *Cx. vishnui*, *Cx. pseudovishnui*, *Cx. gelidus,* *Cx. sitiens,* *Cx. fuscocephela,* * An. subpictus,* *An. hyrcanus, Cx*. *pipiens, Ae. albopictus,* *Ae. japonicas*	Virus of the family Flaviviridae; genus Flavivirus.	Birds, Pigs	Australia, Bangladesh, Burma, Cambodia, China, Guam, India, Indonesia, Japan, Laos, Malaysia, Nepal, North Korea, Pakistan, Papua New Guinea, Phillipines, Russia, Saipan, Singapore, South Korea, Sri Lanka, Taiwan, Thailand, Timor-Leste, Vietnam

## Categorizing LA of EOs Against Different Species

Tables-2 to 9 brief 411 LC_50_ values on LA of different EOs against different species of mosquitoes. Table-2 classifies 152 reports according to LC_50_ of EOs against *Ae. aegypti*. The most potent EOs are *Callitris glaucophylla* and *Piper betle* with LC_50_ of 0.69 and 0.72 µg/mL, respectively. Mentioned EOs could be appropriate for preparing potent herbal larvicides, comparable with synthetic ones.From Table-2, LC_50_ values for 5 EOs are in the range of 1-10 µg/mL: *Auxemma glazioviana, Mammea siamensis, Cinnamomum rhyncophyllum, C. microphyllum,* and *Anacardium occidentale*. LC_50_ of other EOs locate in other 3 groups (i.e. > 10 µg/mL). Table-3 reports the LA of 60 EOs against *Ae. albopictus*. The most potent EOs (with LC_50_< 10 µg/mL) were EOs of *Echinops grijsii* (2.65 µg/mL), *C. microphyllum* (6.20 µg/mL), *C. pubescen* (7.90 µg/mL), *Tetradium glabrifolium* (8.20 µg/mL), *C. mollissimum* (8.80 µg/mL) and *C. impressicostatum* (9.30 µg/mL). Seventeen EOs have LC_50_ between 10-50 µg/mL and remaining have LC_50_ > 50 µg/mL. Table-4 lists LA of 58 EOs against *An. stephensi*. From the details, only LC_50_ of *Kelussia odoratissima* is under 10 µg/mL (~ 5 µg/mL). LC_50_ of *Artemisia dracunculus* (11.36 µg/mL),* Platycladus orientalis*(11.67 µg/mL), *Tagetes patula* (12.08 µg/mL), *Ferulago carduchorum* (12.78 µg/mL), *Chloroxylon swietenia* (14.90 µg/mL) and *Ipomoea cairica* (14.90 µg/mL) are between 10-15 µg/mL. LC_50_ of 19 EOs are in range of 10-50 µg/mL. Table-5 shows reported LA of 16 EOs according to their LC_50_ against *An. subpictus*. Among the plant species, EO of *Ocimum basilicum* with LC_50_ of 9.75 µg/mL is the first in Table-5. EOs of *Eugenia uniflora* and* Heracleum sprengelianum* have similar LC_50_ values (~ 32 µg/mL). LC_50_ of other EOs are > 50 µg/mL. Table-6 summarizes information about LA of some EOs against other species of *Anopheles* such as *An. quadrimaculatus*, *An. gambiae*, *An. anthropophagus*, *An. dirus*, *An. sinensis*, *An. arabiensis*, and *An. marajoara*. Two EOs show excellent LA (i.e., LC_50_ ~1µg/mL): *T. minuta* and *Cananga odorata* against *An. gambiae* and *An. dirus*, respectively. LC_50_ of two other EOs are also worthy to note: *Salvia leucantha* (6.20 µg/mL) against *An. quadrimaculatus* and *Echinops grijsii* (3.43 µg/mL) against *An. sinensis*. Among 66 reports on LA of EOs against *Cx. quinquefasciatus* (Table-7), EO of *Cananga odorata* demonstrates to be the best result with LC_50_ of below 1 µg/mL. After that, LC_50_ of 20 EOs are in the range of 10-50 µg/mL, and LC_50_ of 20 EOs are between 50- 100 µg/mL. LC_50_ of 44 EOs are higher than 50 µg/mL. From Table-8, which summarizes LA of some EOs against *Cx. pipiens,* EOs of *K. odoratissima, Echinops grijsii* and *Pelargonium roseum* show to have LC_50_ at 2.69, 3.43 and 5.49 µg/mL, respectively. They are the most potent EOs against *Cx. pipiens*. Among other EOs, 8 EOs have LC_50_ between 10-50 and others have LC_50_ higher than 50 µg/mL. From Table-9, which briefs the larvicidal activity of different EOs on *Cx. tritaeniorhynchus.* None of the EOs have LC_50_ below 10 µg/mL. However, EOs of *Ocimum basilicum* and *Ipomoea cairica* with LC_50_ ~ 14 can be considered as effective against *Cx. tritaeniorhynchus*. While LC_50_ of other EOs is in range of 36- 136 µg/mL.

**Table 2:** Larvicidal Activity of Essential Oils Against Aedes Aegypti

**No.**	**Plant species**	**Used part(s)**	**LC**_50_ **(µg/mL)**	**Ref**	**No.**	**Plant species**	**Used part(s)**	**LC**_50_ **(µg/mL)**	**Ref**
**1**	*Callitris glaucophylla*	Unclear	0.69	[34]	80	*Piper hostmanianum*	Leaf	54.00	[64]
**2**	*Piper betle*	Leaf	0.72	[17]	81	*Zanthoxylum armatum*	Seed	54.00	[74]
**3**	*Auxemma glazioviana*	Heartwood	2.98	[35]	82	*Croton sonderianus*	Aerial parts	54.50	[56]
**4**	*Mammea siamensis*	Flower	5.90	[36]	83	*Piper aduncum*	Aerial parts	54.50	[75]
**5**	*Cinnamomum rhyncophyllum*	Leaf	6.00	[37]	84	*Carum carvi*	Unclear	54.62	[53]
**6**	*Cinnamomum microphyllum*	Leaf	6.70	[37]	85	*Syzygium lanceolatum*	Leaf	55.11	[76]
**7**	*Anacardium occidentale*	Seed	9.10	[36]	86	*Lippia sidoides*	Leaf	56.00	[57]
**8**	*Piper klotzschianum*	Root	10.00	[38]	87	*Mentha spicata*	Leaf	56.08	[77]
**9**	*Cinnamomum mollissimum*	Leaf	10.20	[37]	88	*Vitex negundo L*	Unclear-	56.13	[22]
**10**	*Cananga odorata*	Flower	10.40	[39]	89	*Salvia officinalis*	Seed	56.90	[42]
**12**	*Cinnamomum impressicostatum*	Leaf	10.70	[37]	90	*Pinus kesiya*	Leaf	57.00	[78]
**14**	*Feronia limonia*	Leaf	11.59	[40]	91	*Lippia pedunculosa*	Unclear	58.00	[18]
**15**	*Citrus sinensis*	Fruit	11.92	[14]	92	*Apium graveolens*	Leaf	59.32	[79]
**16**	*Cinnamomum pubescen*	Leaf	12.80	[37]	93	*Dendropanax morbifera*	Flower	62.32	[80]
**17**	*Piper klotzschianum*	Seed	13.27	[38]	94	*Cordia leucomalloides*	Leaf	63.10	[81]
**18**	*Tagetes patula*	Whole plant	13.57	[41]	95	*Eugenia triquetra*	Aerial parts	64.80	[82]
**19**	*Salvia elegans*	Aerial parts	14.40	[42]	96	*Swinglea glutinosa*	Unclear	65.70	[46]
**20**	*Citrus reticulata*	Fruit	15.42	[43]	97	*Tagetes lucida*	Unclear	66.20	[46]
**21**	*Apium graveolens*	Seed	16.10	[44]	98	*Boswellia ovalifoliolata*	Leaf	66.24	[83]
**22**	*Chloroxylon swietenia*	Leaf	16.50	[45]	99	*Croton nepetaefolius*	Aerial parts	66.40	[56]
**23**	*Cymbopogon flexuosus*	Unclear	17.10	[46]	100	*Origanum scabrum*	Leaf	67.13	[84]
**24**	*Hyptis martiusii*	Unclear	18.20	[47]	101	*Acorus calamus*	Root	67.20	[36]
**25**	*Allium monanthum*	Stem	19.38	[48]	102	*Annona muricata*	Seed	69.25	[36]
**26**	*Lippia sidoides*	Unclear	19.50	[47]	103	*Syzygium aromaticum*	Whole plant	77.00	[85]
**27**	*Piper marginatum*	Stem	19.90	[49]	104	*Eucalyptus citriodora*	Unclear	71.20	[46]
**28**	*Piper marginatum*	Inflorescence	19.90	[49]	105	*Knema globularia*	Seed	72.10	[36]
**29**	*Chloroxylon swietenia*	Stem	20.20	[50]	106	*Capraria biflor*	Leaf	73.39	[86]
**30**	*Citrus sinensis*	Unclear	20.60	[46]	107	*Stemona tuberosa*	Root	75.20	[36]
**31**	*Syzigium aromaticum*	Unclear	21.40	[47]	108	*Samanea saman*	Stem bark	79.20	[36]
**32**	*Cinnamomum scortechinii*	Leaf	21.50	[37]	109	*Croton jacobinenesis*	Leaf	79.30	[87]
**33**	*Ipomoea cairica*	Unclear	22.30	[51]	110	*Tagetes erecta*	Leaf Stem	79.78	[88]
**34**	*Piper marginatum*	Leaf	23.80	[49]	111	*Croton nepetaefolius*	Leaf	84.00	[57]
**35**	*Asarum heterotropoides*	Root	23.82	[52]	112	*Ocimum sanctum*	Aerial parts	85.11	[89]
**36**	*Zanthoxylum limonella*	Unclear	24.61	[53]	113	*Cunninghamia konishii*	Wood	85.70	[90]
**37**	*Psidium guajava*	Leaf	24.70	[54]	114	*Strychnos nux-vomica*	Seed	90.00	[36]
**37**	*Plectranthus mollis*	Whole plant	25.40	[55]	115	*Cunninghamia konishii*	Leaf	91.70	[90]
**39**	*Lippia sidoides*	Aerial parts	25.50	[56]	116	*Syzygium aromaticum*	Bud	92.56	[91]
**40**	*Phyllanthus pulcher*	Leaf & twig	25.80	[36]	117	*Syzygium aromaticum*	Bud	93.56	[14]
**41**	*Croton zehntneri*	Aerial parts	26.20	[56]	118	*Abutilon indicum*	Root	94.20	[36]
**42**	*Anethum graveolens*	Leaf	27.40	[36]	119	*Croton argyrophylloides*	Aerial parts	94.60	[56]
**43**	*Croton zenhtneri*	Leaf	28.00	[57]	120	*Eucalyptus urophylla*	Leaf	95.50	[59]
**44**	*Cryptomeria japonica*	Leaf	28.40	[58]	121	*Cordia curassavica*	Leaf	97.70	[81]
**45**	*Salvia leucantha*	Aerial parts	29.50	[42]	122	*Costus speciosus*	Root	98.50	[36]
**46**	*Citrus hystrix*	Fruit	30.07	[43]	123	*Guarea scabra*	Leaf	98.60	[72]
**47**	*Kaempferia galanga*	Root	30.70	[36]	124	*Nigella sativa L*	Seed	99.90	[92]
**48**	*Eucalyptus camaldulensis*	Leaf	31.00	[59]	125	*Pinus sylvestris*	Needles	100.39	[91]
**49**	*Curcuma zedoaria*	Unclear	31.87	[53]	126	*Croton argyrophyloides*	Leaf	102.00	[57]
**51**	*Eucalyptus grandis*	Leaf	32.40	[60]	127	*Croton sonderianus*	Leaf	104.00	[57]
**52**	*Youngia japonica*	Aerial parts	32.45	[61]	128	*Kadsura heteroclita*	Leaf	111.79	[93]
**53**	*Chenopodium ambrosioides*	Aerial parts	35.00	[62]	129	*Lantana montevidensis*	Leaf	117.00	[68]
**54**	*Murraya exotica*	Leaf	35.80	[63]	130	*Guarea silvatica*	Leaf	117.80	[72]
**55**	*Piper permucronatum*	Leaf	36.00	[64]	131	*Piper gaudichaudianum*	Leaf	121.00	[64]
**56**	*Curcuma aromatica*	Rhizome	36.30	[65]	132	*Croton rhamnifolioides*	Leaf	122.35	[94]
**57**	*Clausena excavata*	Leaf	37.10	[66]	133	*Cymbopogon citratus*	Unclear	123.30	[46]
**58**	*Chamaecyparis formosensis*	Heartwood	38.60	[67]	134	*Syzygium aromaticum*	Flower	124.69	[43]
**59**	*Spondias purpurea*	Leaf	39.70	[54]	135	*Echinophora lamondiana*	Leaf	138.30	[95]
**60**	*Clausena excavata*	Twig	40.10	[66]	136	*Sphaeranthus indicus Linn*	Leaf	140.00	[6]
**61**	*Cinnamomum sintoc*	Leaf	41.10	[37]	137	*Guarea convergens*	Branch	145.10	[72]
**62**	*Apium graveolens*	Unclear	42.07	[53]	138	*Croton tetradenius*	Leaf	152.00	[96]
**63**	*Lippia alba*	Unclear	42.20	[46]	139	*Piper humaytanum*	Leaf	156.00	[64]
**64**	*Lantana camara*	Leaf	42.30	[68]	140	*Cinnamomum cordatum*	Leaf	183.60	[37]
**65**	*Cinnamomum porrectum*	Wood	43.50	[36]	141	*Myrcia ovata*	Leaf	192.10	[54]
**66**	*Zingiber nimmonii*	Rhizome	44.46	[12]	142	*Eugenia piauhiensis*	Leaf	230.00	[97]
**67**	*Blumea eriantha*	Leaf	44.82	[69]	143	*Siparuna camporum*	Leaf	251.00	[97]
**68**	*Zingiber cernuum*	Rhizome	44.88	[21]	144	*Guarea silvatica*	Branch	273.60	[72]
**69**	*Mentha x villosa*	Leaf	45.00	[70]	145	*Lippia gracilis*	Unclear	282.00	[97]
**70**	*Artemisia absinthium*	Leaf	46.33	[71]	146	*Piper aduncum*	Leaf	289.9	[98]
**71**	*Lavandula gibsoni*	Whole plant	48.30	[55]	147	*Psidium myrsinites*	Leaf	292.00	[97]
**72**	*Guarea humaitensis*	Branch	48.60	[72]	148	*Croton argyrophyllus*	Leaf	310.00	[99]
**73**	*Zingiber zerumbet*	Rhizome	48.88	[43]	149	*Mentha piperita L*	Leaf	367.60	[100]
**74**	*Foeniculum vulgare*	Unclear	49.32	[53]	150	*Echinophora lamondiana*	Flower	>125	[95]
**75**	*Plectranthus amboinicus*	Leaf	51.80	[54]	151	*Echinophora lamondiana*	Stem	>125	[95]
**76**	*Eucalyptus nitens*	Leaf	52.83	[73]	152	*Salvia apiana*	Seed	>125	[42]
**77**	*Cananga odorata*	Unclear	52.90	[46]	153	*Myrcia erythroxylon*	Leaf	>1000	[97]
**78**	*Lippia origanoides*	Unclear	53.30	[46]	154	*Xylopia frutescens*	Unclear	>1000	[18]
**79**	*Kaempferia galanga*	Rhizome	53.64	[43]	155	*Xylopia laevigata*	Unclear	>1000	[18]

**Table 3:** Larvicidal Activity of Essential Oils Against *Aedes albopictus*

**No.**	**Plant species**	**Used part(s)**	**LC**_50_ **(µg/mL)**	**Ref**	**No.**	**Plant species**	**Used part(s)**	**LC50** **(µg/mL)**	**Ref**
**1**	*Echinops grijsii*	Root	2.65	[13]	32	*Artemisia absinthium*	Leaf	57.57	[71]
**2**	*Cinnamomum microphyllum*	Leaf	6.20	[37]	33	*Cupressus arizonica*	Leaf	64.80	[107]
**3**	*Cinnamomum pubescen*	Leaf	7.90	[37]	34	*Syzygium lanceolatum*	Leaf	66.71	[76]
**4**	*Tetradium glabrifolium*	Fruit	8.20	[101]	35	*Pinus brutia*	Aerial parts	67.04	[110]
**5**	*Cinnamomum mollissimum*	Leaf	8.80	[37]	36	*Coleus aromaticu*	Leaf	67.98	[111]
**6**	*Cinnamomum impressicostatum*	Leaf	9.30	[37]	37	*Toddalia asiatica*	Root	69.09	[112]
**7**	*Cinnamomum rhyncophyllum*	Leaf	11.80	[37]	38	*Pinus halepensis*	Aerial parts	70.21	[110]
**8**	*Ocimum basilicum*	Leaf	11.97	[102]	39	*Tetraclinis articulata*	Leaf	70.60	[107]
**9**	*Saussurea lappa*	Root	12.41	[103]	40	*Allium macrostemon*	Bulb	72.86	[113]
**10**	*Cinnamomum scortechinii*	Leaf	16.70	[37]	41	*Pinus stankewiczii*	Aerial parts	81.66	[110]
**11**	*Allium tuberosum*	Root	18.00	[104]	42	*Plectranthus barbatus*	Leaf	87.25	[11]
**12**	*Ocimum gratissimum*	Leaf	26.10	[10]	43	*Boswellia ovalifoliolata*	Leaf	89.80	[83]
**13**	*Eucalyptus nitens*	Leaf	28.19	[73]	44	*Syzygium zeylanicum*	Leaf	90.45	[114]
**14**	*Ruta chalepensis*	Leaf	33.18	[105]	45	*Pinus strobus*	Aerial parts	127.98	[110]
**15**	*Eugenia uniflora*	Leaf	33.50	[106]	46	*Foeniculum vulgare*	Leaf	142.90	[115]
**16**	*Chamaecyparis formosensis*	Heartwood	34.90	[67]	47	*Pinus nigra*	Aerial parts	152.65	[110]
**17**	*Cinnamomum sintoc*	Leaf	36.50	[37]	48	*Cinnamomum cordatum*	Leaf	160.80	[37]
**18**	*Cupressus benthamii*	Leaf	37.50	[107]	49	*Helichrysum italicum*	Leaf	178.10	[115]
**19**	*Heracleum sprengelianum*	Leaf	37.50	[108]	50	*Cunninghamia konishii*	Wood	189.50	[90]
**20**	*Cinnamomum osmophloeum*	Leaf	40.80	[109]	51	*Cunninghamia konishii*	Leaf	194.40	[90]
**21**	*Clausena excavata*	Twig	41.10	[66]	52	*Achillea millefolium*	Leaf	211.30	[115]
**22**	*Clausena excavata*	Leaf	41.20	[66]	53	*Hyptis suaveolens*	Leaf	240.30	[116]
**23**	*Chamaecyparis lawsoniana*	Leaf	47.90	[107]	54	*Eucalyptus urophylla*	Leaf	285.80	[59]
**24**	*Cryptomeria japonica*	Leaf	51.20	[58]	55	*Coriandrum sativum*	Fruit	421.00	[117]
**25**	*Cupressus macrocarpa*	Leaf	54.60	[107]	56	*Pinus canariensis*	Aerial parts	>>200	[110]
**26**	*Cupressus sempervirens*	Leaf	54.70	[107]	57	*Pinus pinaster*	Aerial parts	>>200	[110]
**27**	*Eucalyptus camaldulensis*	Leaf	55.30	[59]	58	*Lavandula angustifolia*	Leaf	>250	[115]
**28**	*Juniperus phoenicea*	Leaf	55.50	[107]	59	*Myrtus communis*	Leaf	>250	[115]
**29**	*Zingiber cernuum*	Rhizome	55.84	[21]	60	*Rosmarinus officinalis*	Leaf	>250	[115]
**30**	*Blumea eriantha*	Leaf	56.33	[69]	61	*Artemisia absinthium*	Leaf	57.57	[71]
**31**	*Cupressus torulosa*	Leaf	57.10	[107]	62	*Cupressus arizonica*	Leaf	64.80	[107]

**Table 4:** Larvicidal Activity of Essential Oils Against Anopheles stephensi

**No.**	**Plant species**	**Used part(s)**	**LC**_50_ **(µg/mL)**	**Ref**	**No.**	**Plant species**	**Used part(s)**	**LC**_50_ **(µg/mL)**	**Ref**
**1**	*Kelussia odoratissima*	Aerial parts	4.77	[118]	*30*	*Murraya exotica*	Leaf	56.30	[63]
**2**	*Kelussia odoratissima*	Aerial parts	4.88	[119]	*31*	*Syzigium aromaticum*	Unclear	57.49	[129]
**3**	*Artemisia dracunculus*	Aerial parts	11.36	[8]	*32*	*Zanthoxylum armatum*	Seed	58.00	[74]
**4**	*Platycladus orientalis*	Leaf	11.67	[120]	*33*	*Zhumeria majdae*	Leaf	61.34	[130]
**5**	*Tagetes patula*	Foliage	12.08	[41]	*34*	*Origanum scabrum*	Leaf	61.65	[84]
**6**	*Ferulago carduchorum*	Aerial parts	12.78	[121]	*35*	*Boswellia ovalifoliolata*	Leaf	61.84	[83]
**7**	*Chloroxylon swietenia*	Leaf	14.90	[50]	*36*	*Lavandula gibsoni*	Aerial parts	62.80	[55]
**8**	*Ipomoea cairica*	Unclear	14.90	[51]	*37*	*Origanum vulgare*	Leaf	67.00	[4]
**9**	*Feronia limonia*	Leaf	15.03	[40]	*38*	*Lawsonia inermis*	Leaf	69.40	[131]
**10**	*Chloroxylon swietenia*	Stem	19.00	[50]	*39*	*Cionura erecta*	Root	77.30	[132]
**11**	*Foeniculum vulgare*	Seed	20.10	[122]	*40*	*Cupressus arizonica*	Leaf	79.30	[133]
**12**	*Satureja bachtiarica*	Aerial parts	24.27	[123]	*41*	*Trachyspermum ammi*	Seed	80.77	[134]
**13**	*Bunium persicum*	Seed	27.72	[2]	*42*	*Eucalyptus camaldulensis*	Leaf	89.85	[135]
**14**	*Plectranthus amboinicus*	Leaf	28.37	[124]	*43*	*Coccinia indica*	Leaf	95.30	[136]
*15*	*Citrus aurantium*	Fruit	31.20	[125]	*44*	*Kadsura heteroclita*	Leaf	102.86	[93]
*16*	*Plectranthus mollis*	Aerial parts	33.50	[55]	*45*	*Stachys byzantina*	Leaf	103.29	[131]
*17*	*Achillea kellalensis*	Flower	35.42	[126]	*46*	*Heracleum persicum*	Seed	104.80	[122]
*18*	*Citrus paradisi*	Fruit	35.71	[125]	*47*	*Ajuga chamaecistus tomentella*	Aerial parts	117.72	[137]
*19*	*Anethum graveolens*	Aerial parts	38.80	[127]	*48*	*Coriandrum sativum*	Seed	120.95	[122]
*20*	*Achillea wilhelmsii*	Leaf	39.04	[128]	*49*	*Cedrus deodara*	Leaf	128.04	[131]
*21*	*Zingiber nimmonii*	Rhizome	41.19	[12]	*50*	*Stachys setifera*	Leaf	181.62	[131]
*22*	*Zingiber cernuum*	Rhizome	41.34	[21]	*51*	*Thymus vulgaris*	Leaf	191.33	[131]
*23*	*Blumea eriantha*	Leaf	41.61	[69]	*52*	*Stachys inflata*	Leaf	195.84	[131]

**Table 5:** Larvicidal Activity of Essential Oils Against Anopheles subpictus

**No**	**Plant species**	**Used part(s)**	**LC**_50_ **(µg/mL)**	**Ref**
**1**	*Ocimum basilicum*	Leaf	9.75	[102]
**2**	*Eugenia uniflora*	Leaf	31.08	[106]
**3**	*Heracleum sprengelianum*	Leaf	33.40	[108]
**4**	*Blumea eriantha*	Leaf	51.21	[69]
**5**	*Zingiber cernuum*	Rhizome	51.42	[21]
**6**	*Artemisia absinthium*	Leaf	52.02	[71]
**7**	*Zingiber officinale*	Rhizome	57.98	[140]
**8**	*Coleus aromaticus*	Leaf	60.31	[111]
**9**	*Zhumeria majdae*	Leaf	61.34	[130]
**10**	*Rosmarinus officinalis*	Shoot	64.50	[140]
**11**	*Cinnamomum zeylanicum*	Leaf	71.96	[140]
**12**	*Origanum vulgare*	Leaf	74.14	[4]
**13**	*Cymbopogan citrates*	Leaf	77.24	[140]
**14**	*Boswellia ovalifoliolata*	Leaf	82.26	[83]
**15**	*Syzygium zeylanicum*	Leaf	83.11	[114]
**16**	*Plectranthus barbatus*	Leaf	84.20	[11]

**Table 6:** Larvicidal Activity of Essential Oils Against Other Species of Anopheles

**Plant species**	**Used part(s)**	**Target**	**LC**_50_ ** (µg/mL)**	**Ref**
*Salvia leucantha*	Aerial parts	*An. quadrimaculatus*	6.20	[42]
*Salvia elegans*	Aerial parts	*An. quadrimaculatus*	10.90	[42]
*Salvia officinalis*	Seed	*An. quadrimaculatus*	14.10	[42]
*Ruta chalepensis*	Aerial parts	*An. quadrimaculatus*	14.90	[141]
*Echinophora lamondiana*	Leaf	*An. quadrimaculatus*	26.20	[95]
*Echinophora lamondiana*	Flower	*An. quadrimaculatus*	46.90	[95]
*Echinophora lamondiana*	Stem	*An. quadrimaculatus*	65.60	[95]
*Salvia apiana*	Seed	*An. quadrimaculatus*	>125	[42]
*Tagetes minuta*	Unclear	*An. gambiae*	<1.50	[142]
*Piper capense*	Unclear	*An. gambiae*	34.90	[143]
*Cinnamomum osmophloeum*	Leaf	*An. gambiae*	35.36	[144]
*Plectranthus amboinicus*	Leaf	*An. gambiae*	55.20	[145]
*Blumea martiniana*	Aerial parts	*An. anthropophagus*	46.86	[146]
*Artemisia gilvescens*	Unclear	*An. anthropophagus*	49.95	[147]
*Cananga odorata*	Flower	*An. dirus*	<1	[39]
*Echinops grijsii*	Root	*An. sinensis*	3.43	[13]
*Juniperus procera*	Unclear	*An. arabiensis*	14.42	[148]
*Piper aduncum*	Aerial parts	*An. marajoara*	50.90	[75]

**Table 7:** Larvicidal Activity of Essential Oils Against Culex quinquefasciatus

**No.**	**Plant species**	**Used part(s)**	**LC**_50_ ** (µg/mL)**	**Ref**	**No.**	**Plant species**	**Used part(s)**	**LC**_50_ ** (µg/mL)**	**Ref**
**1**	*Cananga odorata*	Flower	<1	[39]	*34*	*Boswellia ovalifoliolata*	Leaf	72.47	[83]
**2**	*Mentha longifolia*	Unclear	17.00	[149]	*35*	*Pimenta dioica*	Fruit & berry	77.20	[153]
**3**	*Mentha suaveolens*	Unclear	17.00	[149]	*36*	*Origanum vulgare*	Leaf	80.35	[4]
**4**	*Achillea kellalensis*	Flower	21.79	[126]	*37*	*Peumus boldus*	Leaf	82.14	[157]
**5**	*Tagetes patula*	Foliage	22.33	[41]	*38*	*Zhumeria majdae*	Leaf	88.51	[130]
**6**	*Feronia limonia*	Leaf	22.49	[40]	*39*	*Mentha spicata*	Unclear	92.00	[149]
**7**	*Satureja montana*	Aerial parts	25.60	[150]	*40*	*Pelargonium graveolens)*	Aerial parts	98.40	[150]
**8**	*Pimpinella anisum*	Fruit	26.10	[151]	*41*	*Hyssopus officinalis*	Aerial parts	99.50	[150]
**9**	*Tanacetum persicum*	Aerial parts	28.53	[126]	*42*	*Ravensara aromatica*	Leaf	101.40	[153]
**10**	*Plectranthus mollis*	Whole plant	29.50	[55]	*43*	*Anthemis nobilis*	Flower	108.70	[153]
**11**	*Rosmarinus officinalis*	Stem & Leaf	30.60	[152]	*44*	*Rosmarinus officinali*	Flowering herb	111.10	[153]
**12**	*Thymus vulgare*	Flowering top	32.90	[153]	*45*	*Nepeta cataria*	Flowering top	112.40	[153]
**13**	*Satureja hortensis*	Flowering top	36.10	[153]	*46*	*Mentha aquatica*	Unclear	118.00	[149]
**14**	*Murraya exotica*	Leaf	43.20	[63]	*47*	*Lavandula angustifolia*	Flower	121.60	[153]
**15**	*Thymus satureoides Boiss*	Herb	43.60	[153]	*48*	*Kadsura heteroclita*	Leaf	121.97	[93]
**16**	*Satureja bachtiarica*	Aerial parts	44.96	[123]	*49*	*Syzygium aromaticum*	Buds	124.42	[91]
**17**	*Zingiber nimmonii*	Rhizome	48.26	[12]	*50*	*Cannabis sativa*	Herb	127.30	[153]
**18**	*Zingiber cernuum*	Rhizome	48.44	[21]	*51*	*Salvia sclarea*	Flower	127.50	[153]
**19**	*Blumea eriantha*	Leaf	48.92	[69]	*52*	*Pinus sylvestris*	Needles	128.00	[91]
**20**	*Zanthoxylum armatum*	Seed	49.00	[74]	*53*	*Sphaeranthus indicus*	Leaf	130.00	[6]
**21**	*Pinus nigra*	Twig	49.80	[150]	*54*	*Pelargonium roseum*	Leaf	130.30	[153]
**22**	*Artemisia absinthium*	Unclear	50.57	[71]	*55*	*Nigella sativa*	Seed	141.70	[92]
**23**	*Zingiber officinalis*	Rhizome	50.78	[154]	*56*	*Erigeron canadensis*	Herb	141.90	[153]
**24**	*Lavandula gibsoni*	Whole plant	54.70	[55]	*57*	*Juniperus communis*	Berry & twig	164.30	[153]
**25**	*Ipomoea cairica*	Unclear	58.90	[51]	*58*	*Laurus nobilis*	Leaf	167.90	[153]
**26**	*Syzygium lanceolatum*	Leaf	60.01	[76]	*59*	*Amyris balsamifera*	Wood	170.70	[153]
**27**	*Pinus kesiya*	Leaf	62.00	[78]	*60*	*Ocimum basilicum*	Leaf	171.60	[153]
**28**	*Mentha spicata*	Leaf	62.62	[77]	*61*	*Citrus aurantium*	Flower	179.80	[153]
**29**	*Psoralea corylifolia*	Seed	63.38	[155]	*62*	*Tanacetum vulgare*	Flowering top	186.60	[153]
**30**	*Pulegium vulgare*	Unclear	64.00	[149]	*63*	*Zingiber cassumunar Roxb*	Root	202.30	[153]
**31**	*Aloysia citrodora*	Leaf	65.60	[150]	*64*	*Melaleuca alternifolia*	Leaf	204.10	[153]
**32**	*Blumea mollis*	Leaf	71.71	[156]	*65*	*Santalum album*	Heartwood	225.30	[153]
**33**	*Origanum scabrum*	Leaf	72.45	[84]	*66*	*Polygonum hydropiper*	Leaf	243.00	[139]

**Table 8:** Larvicidal Activity of Essential Oils Against Culex pipiens

**Plant species**	**Used part(s)**	**LC**_50_ ** (µg/mL)**	**Ref**
*Kelussia odoratissima*	Aerial parts	2.69	[119]
*Echinops grijsii*	Root	3.43	[13]
*Pelargonium roseum*	Leaf	5.49	[158]
*Platycladus orientalis*	Leaf	18.60	[2]
*Bunium persicum*	Seed	20.61	[2]
*Asarum heterotropoides*	Root	21.07	[52]
*Thymus teucrioides*	Aerial parts	23.17	[159]
*Citrus limon*	Lemon	30.14	[160]
*Thymus leucospermus*	Aerial parts	34.26	[159]
*Citrus aurantium*	Bitter orange	39.81	[160]
*Oenanthe pimpinelloides*	Aerial parts	40.26	[161]
*Citrus sinensis*	Sweet orange	51.50	[160]
*Geranium maculatum*	Unclear	57.28	[162]
*Bupleurum fruticosum*	Aerial parts	64.68	[161]
*Conopodium capillifolium*	Aerial parts	68.50	[161]
*Heracleum sphondylium*	Aerial parts	77.41	[161]
*Citrus bergamia*	Unclear	81.45	[162]
*Seseli montanum*	Aerial parts	86.60	[161]
*Eleoselinum asclepium*	Aerial parts	96.96	[161]
*Hypericum tomentosum from Tbarka*	Aerial parts	102.82	[163]
*Hypericum tomentosum from Fernana*	Aerial parts	125.26	[163]
*Hypericum humifusum*	Aerial parts	156.80	[163]
*Hypericum perforatum*	Aerial parts	194.70	[163]

**Table 9:** Larvicidal Activity of Essential Oils Against Culex tritaeniorhynchus

**Plant species**	**Used part (s)**	**LC**_50_ ** (µg/mL)**	**Ref**
*Ocimum basilicum*	Leaf	14.01	[102]
*Ipomoea cairica*	Unclear	14.80	[51]
*Eugenia uniflora*	Leaf	36.35	[106]
*Heracleum sprengelianum*	Leaf	40.90	[108]
*Zingiber cernuum*	Rhizome	60.20	[21]
*Blumea eriantha*	Leaf	61.33	[69]
*Artemisia absinthium*	Unclear	62.16	[71]
*Syzygium lanceolatum*	Leaf	72.24	[76]
*Coleus aromaticus*	Leaf	72.70	[111]
*Origanum scabrum*	Leaf	78.87	[84]
*Origanum vulgare*	Leaf	84.93	[4]
*Plectranthus barbatus*	Unclear	94.34	[11]
*Boswellia ovalifoliolata*	Leaf	97.95	[83]
*Syzygium zeylanicum*	Leaf	97.96	[114]
*Zingiber officinale*	Rhizome	98.83	[140]
*Rosmarinus officinalis*	Shoot	115.38	[140]
*Cinnamomum zeylanicum*	Bark	124.70	[140]
*Cymbopogan citrates*	Leaf	136.58	[140]

## Potent EOs in Terms of LA

Reviewing Tables-2 to 9, some EOs demonstrate proper LA against at least two species, thus, may be suggested as attractive candidates for preparing EO-based larvicides (Table-10). For instance, LC_50_ of *Echinops grijsii* is ~ 3 µg/mL against three species: *Cx. pipiens*, *An. sinensis* and *Ae. albopictus*. EO of *Cananga odorata* is another candidate with LC_50_ ~ 1 µg/mL against *Cx. quinquefasciatus* and *An. dirus* and LC_50_ of 10 µg/mL against *Ae. aegypti*. EO of *K. odoratissima* with LC_50_ of 2 and 4 µg/mL against *Cx. pipiens* and *An. stephensi* respectively, could also be considered as a potent larvicide.Besides mentioned EOs, the LA of 4 EOs is comparable with classic larvicide (i.e., ~ 1 µg/mL). LC_50_ of *Callitris glaucophylla* and *Piper betle* against *Ae. aegypti* are 0.69 and 0.72 µg/mL, respectively. *Cananga odorata* show LC_50_ < 1 µg/mL against both of *Cx. quinquefasciatus* and *An. Dirus.* EO of *T. minuta* has excellent LA against *An. gambiae* (LC_50_ < 1.5 µg/mL).

**Table 10:** Potent Essential Oils as Larvicide Against at Least 2 Species of Mosquitoes

**Plant species**	**Target**	**LC**_50_ ** (µg/mL)**	**Ref**
*Ocimum basilicum*	*Cx. tritaeniorhynchus*	14.01	[102]
	*An. subpictus*	9.75	
*Kelussia odoratissima*	*Cx. pipiens*	2.69	[118, 119]
	*An. stephensi*	4.77	
	*An. stephensi*	4.88	
*Echinops grijsii*	*Cx. pipiens*	3.43	[13]
	*An. sinensis*	3.43	
	*Ae. albopictus*	2.65	
*Cananga odorata*	*Cx. quinquefasciatus*	<1	[39]
	*An. dirus*	<1	
	*Ae. aegypti*	10.40	
*Cinnamomum microphyllum*	*Ae. albopictus*	6.20	[37]
	*Ae. aegypti*	10.70	
*Cinnamomum pubescen*	*Ae. albopictus*	7.90	[37]
	*Ae. aegypti*	10.20	
*Cinnamomum impressicostatum*	*Ae. albopictus*	9.30	[37]
	*Ae. aegypti*	10.70	
*Cinnamomum rhyncophyllum*	*Ae. albopictus*	11.80	[37]
	*Ae. aegypti*	6.00	

## Advantages of EOs as Larvicides

To control mosquito-borne diseases such as malaria, world health organization (WHO) recommends using larvicides; nowadays using in 55 countries around the worlds [164]. Continuous use of synthetic larvicides such as malathion and temephos along with environmental pollution, lead to occurring resistance in a various population of mosquitos such as *Ae. aegypti, Cx. pipiens* and *An. stephensi* [165-168].Furthermore, many reports may be found about the impacts of the larvicides against non-target species. For instance, dichlorvos and tetraethyl pyrophosphate (belonging to organophosphates larvicides) and carbofuran (carbamates) have an effect on acetylcholinesterase in some species of fishes including *Arapaima gigas*, *Rachycentron canadum*, *Oreochromis niloticus*, and *Electrophorus electricus* [169]. In another study, sides effects of 2 other larvicides including Temephos and Novaluron against 10 species of aquatic insect families and copepods have been evaluated. It was revealed that their impact on Veliidae, Odonata, Dytiscidae are significantly higher than that of other [170]. Oudemans (*Amblyseius cucumeris*) is a crucial predator of mites of tetranychid while two other common pesticides, i.e., Bifenthrin and Malathion posed an extremely effect on this beneficial non-target arthropod [171].EOs are naturally extracted aroma compounds with broad applications such as flavoring additives, medicines, antioxidants, antifungal/bacterial and also larvicides [172-177]. In the past decade, EO based formulation have been suggested as alternative sources for control of mosquitoes to be used as larvicides [8, 127]. They offer advantages such as biodegradability, negligible effects on non-target specious and environment [101, 178]. Besides, resistance against larvicides is observed when a single active agent is used compared with those having multi-components, thus by using EOs, decreases the risk of occurring resistance in mosquito populations [14-16]. EOs are mixtures of many constituents such as flavonoids, alkaloids, and monoterpenes [179, 180]. Modes of action of mentioned constituents are different, for instance, main sites action of alkaloids and monoterpenes are Na-K-ATPase or Na+ and K+ channels [19, 181, 182], while flavonoids target acetylcholinesterase [183]. Synergistic effects of constituents of some EOs are nowadays well-known when they are used as anti-fungal or anti-bacterial agents [184, 185]. Types of synergism also reported in larvicidal studies, e.g., larvicidal activities (LC_50_) of EOs of *Syzygium aromaticum* and *K. odoratissima* (57.49 and 4.77 µg/mL, respectively) significantly better than their major constituents, i.e., Eugenol (86.96 µg/mL) and Z-ligustilide (8.73 µg/mL) against *An. stephensi* [118, 129].

## Conclusion

In this paper, mosquito-borne diseases have been reviewed. Previous studies about LA of EOs against different species of mosquitoes including *Aedes*, *Anopheles*, and *Culex* were investigated. For the first time, 411 LC_50_ were ranked against each species, separately. LC_50_ of 4 EOs are ~ 1µg/mL, including *Callitris glaucophylla* and * Piper betle* against *Ae. aegypti*, *T. minuta* against *An. gambiae*, and *Cananga odorata* against *Cx. quinquefasciatus* and *An. dirus*. The potency of mentioned EOs is comparable with synthetic larvicides, while simultaneously having some advantages such as reducing the chance of resistance and minimum sides’ effects on non-target species. Thus, it could be considered as candidates for preparing botanical larvicides.

## Acknowledgment

This study was supported by Tehran University of Medical Sciences & Health Services (grant No. 95-01-87-31860).

## Conflict of Interest

There is no conflict of interest to the authors.
